# Evaluation of Transition to Electronic Prescriptions in Turkey: Perspective of Family Physicians

**DOI:** 10.15171/ijhpm.2018.89

**Published:** 2018-09-23

**Authors:** Sinan Bulut, Ahmet Yıldız, Sıdıka Kaya

**Affiliations:** ^1^Department of Health Care Management, Faculty of Health Sciences, Hitit University, Çorum, Turkey.; ^2^Program of Health Institutions Management, Vocational School of Health Services, Batman University, Batman, Turkey.; ^3^ Department of Health Care Management, Faculty of Economics and Administrative Sciences, Hacettepe University, Ankara, Turkey.

**Keywords:** Family Physicians, Electronic Prescribing, Health Technology Assessment, Turkey

## Abstract

**Background:** One of the most important steps of the health transformation program involves the application of electronic prescriptions (e-prescriptions) in health services. Information technologies are highly important in generating e-prescriptions, which can be described as a document produced by authorized personnel electronically, containing written information and instructions regarding a patient’s medication and its usage. E-prescribing has become increasingly applied in recent years as a contributing application to prescribers and patients. The aim of this study was to determine the level of satisfaction of family physicians providing primary care in Turkey regarding the application of e-prescriptions, and reveal the related positive effects and problems encountered in the first months of implementation of e-prescribing.

**Methods:** A questionnaire with eight questions was sent to e-mails of all family physicians working in Turkey in May 2013. A total of 1564 family physicians filled in the questionnaire form and sent it back by e-mail. The responses to openended questions were evaluated by content analysis.

**Results:** It was observed that the most frequently indicated advantages of the application of e-prescriptions were speeding up the prescription process and saving time (36.6%). The most commonly reported problems regarding the application of e-prescriptions were found to be system-induced problems (26.5%) and internet problems (19.9%). In addition, the mean score of satisfaction of the family physicians who did not report problems with the application of e-prescriptions was higher than that of those who reported having problems with it. In the study, 77.8% of the family physicians were satisfied with the application of e-prescriptions.

**Conclusion:** Although some problems were reported regarding the application of e-prescriptions in the first months of the application, family physicians participated in the study were found to be satisfied with the application of e-prescriptions, and identified positive effects on their work and processes.

## Background

Health information technology includes a variety of technologies that enable the management and transfer of information for patients, service providers, insurers, payment institutions and all other groups related to health and healthcare.^1^ The utilization of information technologies in healthcare services can make potential contributions to enhancing service quality, safety, efficiency and reducing costs both for patients and service providers.^2,3^ Although such contributions have been reported, technology usage by physicians and hospitals is still at a low level.^2^

In Turkey, the Health Transformation Program, which includes initiatives for increasing the utilization of information technologies in the provision of health services, aims to “put into practice the e-transformation project in the field of health” and, in this context, to promote the constitution, standardization, and classification of information systems, and the integration and active use of data gathered from different institutions.^4^

Electronic prescription (e-prescription) is one of the important steps taken to use information systems in the field of health in terms of facilitating communication between institutions in prescription processes, increasing patient safety and satisfaction. Technology has the advantage of increasing people’s work performance and people can be stronger with technology than they are alone. Thus human errors can decrease.^5^ In addition, information technology plays a key role in providing better and safer care, and transformation of health services.^6^ That is why information technologies are highly important in generating e-prescriptions, which can be described as a document produced by authorized personnel electronically, containing written information and instructions regarding a patient’s medication and its usage.

Despite the potential advantages, launching new applications such as e-prescriptions does not mean that it can be successfully implemented. Human factors can play an important role in the success of the new technology, and user satisfaction is one of them. It is important for users to be satisfied with the new system to implement the system successfully.^7,8^

Electronic systems for medicine prescribing and administration are widely used in the United States. This is probably due to the need for costing of medication administration, in an insurance-based health system, and the need for risk management to reduce clinical risk to a minimum, to optimise audit trails in a highly litigious society, and to improve the quality of patient care.^9,10^

Electronic prescribing, which is beginning to show more and more positive effects in the United States, began to spread later in Europe. The e-prescription was first used routinely in Europe in 1983 in Sweden. The functionality of the e-prescription has expanded over time. With a national postal box system launched in 2004, patients became able to take medicines from any pharmacist and to access their prescriptions through an online portal. Like Sweden and Denmark, it can be seen among countries that have adopted health information technology in the early period and pioneered the use of this technology.^11^ In Denmark, the e-prescription system began in 1994 with the central e-Health organization Medcom, which sets the standards for e-prescriptions and ensures stakeholder compliance. In Denmark, over 99% of total prescriptions are e-prescription, while in Sweden this rate is over 90%.^11^ Creating the required standards, gaining agreement to use the standards and funding implementation of the standards are play a key role to success e-prescription in Denmark.^12^ In the United Kingdom, there is a great difference in terms of e-prescription between primary and secondary healthcare services. In primary healthcare, the electronic prescription system is relatively well-structured. There are 2 periods in e-prescription practice in UK primary healthcare. The first period is the period when the pilot was initiated in 2005 and the second is the period in which the electronic transmission is started in 2012. In the second period, 99% of public pharmacies started using e-prescriptions.^11^ Electronic prescription (e-prescription) systems have become increasingly widespread in developing countries in recent years.^13-15^ Health systems where there is a clear hierarchical structure and where healthcare is managed by a single authority are more successful in e-prescription adoption. In addition, each country’s specific legislation, regulation and stakeholder engagement play an important role in the development of e-prescribing practices.^14^ In order for e-prescription applications to work well, it is important to set standards for data entry fields in e-prescription software. These standards will ensure that the prescription is created in a clear and complete manner and will prevent physicians from filling data entry fields in different ways.^16^

E-prescription is a software application that shares patients’ information over a network that is open to the common use of health service providers, and allows the storage, utilization by relevant staff, and transmission of this information through writing in an electronic environment.^17^ Additionally, e-prescribing can be described as producing a prescription by entering data (into a computer or a mobile portable device for instance) about the patient and medication in the automatic data entry system, instead of writing the prescription on paper manually. According to the Centre of Medicare and Medicaid services in the United States, *“E-prescribing means the transmission, using electronic media, of prescription or prescription-related information between a prescriber, dispenser, pharmacy benefit manager, or health plan, either directly or through an intermediary, including an e-prescribing network.”*^18^ E-prescription is defined by the Social Security Institution (SSI) in Turkey as “*prescriptions generated by physicians on the systems of the health service providers in accordance with the definitions and announcements of the SSI, and are electronically recorded in the MEDULA [Medikal Ulak] system and given the electronic prescription number.*”^19^

In the process of e-transformation in the field of health in Turkey, the first preparation e-prescriptions was conducted in 2011. Subsequently, studies on the application of e-prescriptions intensified and in 2012, a pilot implementation was initiated in 3 provinces. Following this pilot implementation, on July 1, 2012, the e-prescription system used by the main repayment institution, the SSI, was extended to the whole country, and on January 15, 2013 e-prescribing became compulsory except in certain exceptional circumstances.^20^ The introduction of e-prescriptions has been successful, as indicated by the fact that in the first half-period of the implementation 65% of all prescriptions were generated electronically, and 105 200 physicians obtained institutional passwords in order to create e-prescriptions.^21^

The SSI has been conducting the application of e-prescriptions on the system of MEDULA (Also see Figure), which is an integrated system including the General Health Insurance (GHI) and healthcare facilities in order to gather billing data electronically and make service payments without interfering with internal processes.^21^ For example, before the e-prescription application, SSI required that the paper prescriptions be delivered to the institution within a certain period of time, checked if the prescription was signed and if the medications were appropriate. With the application of e-prescriptions, however, these procedures can be easily carried out through the system.

**Figure:**
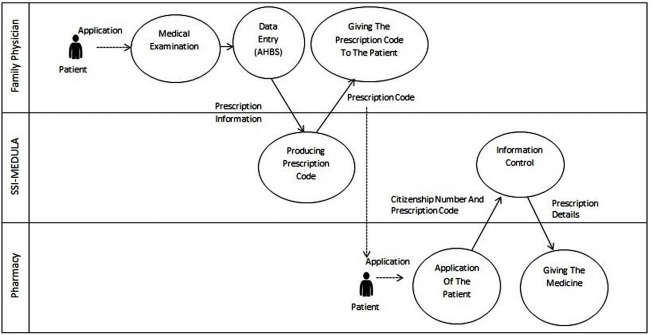
E-prescription Process in Medikal Ulak (MEDULA) System in Turkey.

The system involves the use of patient information and is available to physicians, pharmacies, and hospitals. The system ensures the flow of information without interfering with the processes of health institutions. Pharmacies, health centers, diagnostic and treatment centers and public and private hospitals have been integrated into MEDULA via web services. MEDULA system which provides accurate and practical communication between health service providers and SSI acts jointly with the GHI, the Central Population Affairs System (Mernis) and the Ministry of Health.^22^ The integration of the MEDULA system with other institutions also provides the physician with information regarding the patient. By this means, when the physician enters a patient’s identification number into the system for e-prescription, the patient’s identification information from the Mernis system appears on the screen. In addition, the physician can see the information regarding the patient’s social security on the screen.

Family physicians, who are primary healthcare service providers, have been prescribing electronically except in accepted exceptional circumstances (prescriptions written when providing mobile or home healthcare services, and when the MEDULA or healthcare service provider systems do not work).^23^ Family physicians can create e-prescriptions on the family practice information system (AHBS), which is compatible with the MEDULA system and also provided by private companies, by obtaining an institutional physician password provided by the SSI. Using this password, family physicians can access the reports of any patient, their reported or unreported medicines, test results, medical information, and repayment information on the MEDULA system.

The e-prescription process is carried out in a system involving health service providers, Social Security Institution and pharmacies. In the process of healthcare service providing, which starts with the patient’s visit to the family physician, the physician logs into AHBS, using their institutional password obtained from the SSI. E-prescriptions are generated by accessing the patient’s information in MEDULA using the citizenship number of the patient. A code for the generated prescription is provided by the MEDULA system and this code is given to the patient by the physician. The patient can then obtain their medicine from any pharmacy of their choice (Also see Figure). While family physicians are the first step in the e-prescription process at the primary level, SSI forms the second step by generating, transmitting and controlling the prescription code, and pharmacies form the last step of the system as users of the e-prescription codes.

This study, which was carried out in the first months of the implementation of the e-prescription system in Turkey, intended to provide information about the functionality of the implementation to the decision makers in Turkey and in other countries planning to apply e-prescription systems. Specific aims of this study are to:

1. Determine the positive effects of e-prescriptions on the procedures of family physicians that provide primary healthcare services,
2. Determine the problems involved in e-prescription writing,
3. Determine the level of satisfaction of family physicians with the application of e-prescriptions.

## Methods

### Data Collection Tool

A questionnaire developed specifically for this research was used in the study. To begin with, 30 family physicians were interviewed face-to-face about the e-prescribing system they used; the convenience of the system, the challenges they have experienced and their satisfaction with the system. A data collection tool was generated by the authors using these interviews as well as SSI communiqué on healthcare practices,^24^ and informational correspondences for the use of e-prescribing written by the SSI and Public Health Agency of Turkey. Since the interviewees stated that number of questions should be kept minimum due to the heavy workload of family physicians, the questionnaire was limited to only 8 questions. Then the questionnaire was pilot tested and participants found the questions were understandable and feasible. In addition to the questions related to age, years of experience as a physician, number of e-prescriptions written daily, and time taken to write an e-prescription, the following questions were also included a 5-point Likert-type question to determine the satisfaction level of physicians with the application of e-prescriptions, and 2 questions to determine the positive effects of e-prescriptions on physicians’ processes and whether problems with e-prescriptions were experienced, “Has the application of e-prescriptions had positive effects on your work and processes? If yes, what are these?” and “Are you having problems with the application of e-prescriptions? If yes, what are these?” The necessary permissions were obtained from the Ministry of Health, Public Health Institution of Turkey in order to conduct the study.

### Population and Sampling

In May 2013, when the research was conducted, 20 450 family physicians were working in Turkey. No sample was selected and it was aimed to include as many family physicians as possible in the study. Data were collected using e-mails of family physicians. The questionnaire form was forwarded once to all family physicians via e-mail and responses were archived to be evaluated. A total of 1534 family physicians (7.5%) participated in the study. It has been tested whether the participants represent the family practitioner population by using age and sex distributions of family physicians. According to the Ministry of Health, 68% (13 906) of the family physicians working in Turkey were male and 32% (6544) were female as of the date of the research. 70.3% (1078) of the family physicians participating in the survey were male and 29.7% (456) were female. No statistically significant difference was found between the rates (χ^2^= 3.999; *P* > .05). In addition, according to the data obtained from the Ministry of Health, the average age of the family physicians as of the date of the research was 40.62. The average age of family physicians participating in the survey was 41. There was no statistically significant difference between the averages. (t= 1.926; *P* > .05). Accordingly, it can be said that the participants represent the family practitioner population in terms of age and gender distribution.

### Data Analysis

Professional and demographic information and the level of satisfaction with the application of e-prescriptions of the family physicians in the research were analyzed using descriptive statistics such as mean, standard deviation, frequency, and percentage. Significance testing (*t* test) of the difference between 2 means was conducted in order to evaluate whether the level of satisfaction with the application of e-prescriptions differed or not according to the “yes-no” answers of family physicians to the questions “Has the application of e-prescriptions had positive effects on your work and processes?” and “Are you having problems with the application of e-prescriptions?” Each response given by physicians to these questions were evaluated and content analysis was conducted. Responses that express the same problem or positive effect were grouped under the same heading. In this way, all the answers were examined and the positive effects and problems were tabulated under certain headings. Microsoft Excel was used to classify the answers to the open-ended questions related to the positive effects and the problems experienced in relation to the application of e-prescriptions. The Statistical Package for the Social Science for Windows (SPSS 20.0) was used to perform the statistical analyses.

## Results

Family physicians’ age, years of experiences as a physician, number of e-prescriptions written per day, time taken to write an e-prescription, and level of satisfaction related to the application of e-prescriptions are shown in Table 1. When examining the ages of all 1534 family physicians participating in the research, it can be seen that family physicians’ mean age was 40 years. The mean years of experiences as a physician was nearly 3 years. In terms of the number of e-prescriptions written per day, the mean number was approximately 50 prescriptions. The mean time taken by family physicians to write an e-prescription was nearly 3 minutes. Regarding the level of satisfaction with the application of e-prescriptions, a significant percentage was satisfied (77.8%) and a small percentage was not (5.1%). The mean score for the level of satisfaction was calculated as 4.15 ± 0.943 and this score was evaluated as high.

**Table 1:** Characteristics of the Family Physicians Participated in the Study (N = 1534)

		**N**	**Percent**	**Mean ± SD (Max-Min)**
Age (y)	≤35	394	25.7	41.00 ± 7.72 (66**-**24)
	36-45	666	43.4	
	≥46	574	30.9	
Time Working as Physician (y)	≤2	355	23.2	3.33 ± 1.39 (7**-**1)
	3-4	839	54.8	
	≥5	338	22.1	
Number of e-prescriptions written per day	≤35	367	24.0	47.20 ± 16.22 (120**-**6)
	36-50	700	45.8	
	≥51	462	30.2	
Time taken to write an e-prescription (min)	≤2	797	52.3	2.86 ± 2.12 (15**-**0.1)
	3-4	420	27.5	
	≥5	308	20.1	
Level of satisfaction^a^	1	33	2.2	4.15 ± 0.94 (5**-**1)
	2	45	2.9	
	3	247	16.1	
	4	536	34.9	
	5	658	42.9	

Abbreviation: SD, standard deviation. ^a^1 = Absolutely not satisfied,…, 5 = Absolutely satisfied.

It can be seen that the number of physicians who answered “yes” to the question “Has the application of e-prescriptions had positive effects on your work and processes?” is nearly 4 times higher than those who said “no” (Table 2). The level of satisfaction with the application of e-prescriptions of those who said “yes” was nearly 1 point higher than that of those who said “no,” with the difference being statistically significant (*P* < .001). Similarly, the number of those who said “yes” to the question “Are you having problems with the application of e-prescription?” was higher than those who said “no.” The level of satisfaction with the application of e-prescriptions of those who said “yes” to this question was nearly 1 point lower than of that of those who said “no,” with the difference also being statistically significant (*P* < .001). The higher number of physicians answering “yes” to both questions suggests that although family physicians have problems with the application of e-prescriptions they also think that e-prescriptions have positive effects. This demonstrates that e-prescription practice is beneficial but open to improvements. The high level of satisfaction of family physicians who report no problems suggests that satisfaction would generally increase if the problems with the application of e-prescriptions were eliminated.

**Table 2:** Distribution of Family Physicians’ Level of Satisfaction With Application of E-prescriptions According to Answers to Questions “Has the Application of E-prescriptions Had Positive Effects on Your Work and Processes?” and “Are You Having Problems With the Application of E-prescriptions?”

		**n**	**Level of Satisfaction**		***t***	***P***
			**Mean**	**SD**		
Has the application of e-prescriptions had positive effects on your work and processes?	Yes	1229	4.31	0.80	11.892	<.001
	No	290	3.45	1.17		
Are you having problems with the application of e-prescriptions?	Yes	987	3.89	0.98	-17.109	<.001
	No	532	4.61	0.65		

Abbreviation: SD, standard deviation.

The positive effects of the application of e-prescriptions reported by the participants are shown in Table 3. Of the 1534 family physicians who participated in the survey, 1020 (66.49%) stated at least one positive effect of the application of e-prescriptions. A total of 1975 statements about the positive effects of the application of e-prescriptions on the work and processes of family physicians were collected. These statements were gathered under 5 groups and 18 subgroups. Most of the surveyed physicians (79.4%) stated that e-prescription application saves time, paper and toner. One in 4 physicians pointed out that e-prescribing facilitated prescription writing. In addition, it was expressed that e-prescription application increases the quality and reliability of service, increases patient satisfaction and prevents conflicts between physician-patient and pharmacist about prescribing.

**Table 3:** Family Physicians’ Answers to Question “Has the Application of E-prescriptions Had Positive Effects on Your Work and Processes?”

	**Positive Effects**	**n**	**Percent** ^a^
	A- Providing savings	1218	79.4
1	Speeding up prescription writing and saving time	561	36.6
2	Paper saving	438	28.6
3	Toner saving	219	14.3
	B- Facilitating prescription writing	390	25.4
4	Removing handwriting and simplifying prescription writing	171	11.1
5	Being able to see former medicines and reports provided to patient, and information related to patient on the system, and simplifying following-up the patient	123	8.0
6	Simplifying generation of prescription and allowing adding explanation to prescription	63	4.1
7	Being able to see the medicine, dosage, equivalents, and prices on the system and providing convenience to physician in determining the medicine to be prescribed	24	1.6
8	Enabling prescription writing even in external environments (mobile services) where internet access is provided	9	0.6
	C- Increasing service quality and reliability	231	15.1
9	Reducing prescription writing errors, e-prescriptions being legible, exact, and complete, providing convenience to pharmacist, and reducing the errors of providing incorrect medicine or dosage	143	9.3
10	E-prescriptions being safe, records being reliable, and no alterations to be made on prescription by others	88	5.7
	D- Facilitating patient’s process to obtain medicine and increasing patient satisfaction	62	4.0
11	Simplifying patient’s process to obtain medicine	18	1.2
12	Increasing patient satisfaction	15	1.0
13	Reducing patient waiting time	11	0.7
14	Creating the perception of more contemporary, technological, and higher-quality service	10	0.7
15	Eliminating situations such as loss or tearing of prescription	8	0.5
	E- Preventing physician-patient-pharmacist conflict	52	3.4
16	Reducing arguments over prescription writing with patient	27	1.8
17	Reducing arguments over prescription writing between physician and pharmacist	25	1.6
	F- Other	22	1.4
18	Other	22	1.4
	Total	1975	

^a^Since some physicians gave more than one answer to this question, the total of the percentage exceeds 100.

Of the 1534 family physicians who participated in the study, 966 (62.97%) stated that they had experienced at least one problem with the application of e-prescriptions. A total of 1756 statements were collected about the problems experienced in the application of e-prescriptions. These statements were gathered under 4 groups and 23 subgroups (Table 4). An important part of the complaints consisted of system-induced problems. More than a quarter (26.5%) of the family physicians in the research complained about system-induced problems such as slow working of the system, stopping of the system from time to time. Internet-related problems were also important. The implementation of e-prescription through the internet network requires the establishment of a robust internet infrastructure. Interrupting or slowing down the connection to the Internet hinders e-prescription application. Apart from these, problems with regard to prescription writing (stemming from reasons such as absence of some desired medicines on the system or not being able to prescribe them; not being able to correct, add, delete after the prescription has been sent; absence of some medicines, medicine dosages or diagnosis in the system) were among the most common complaints.

**Table 4:** Family Physicians’ Answers to Question “Are You Having Problems With the Application of E-prescriptions?”

	**Problems**	**n**	**Percent** ^a^
	A- Infrastructure, system (software) and internet related problems	1148	74.8
1	System-induced problems	407	26.5
2	Internet problems	304	19.8
3	Not being able to obtain prescription code	231	15.1
4	Loss of time, waiting due to internet interruption or system failure	179	11.7
5	Problems with barcode	21	1.4
6	Power cut	16	1.0
7	Failure in functioning of computer (hardware)	6	0.4
8	Inadequacy of infrastructure	5	0.3
	B- Problems with prescription writing	389	25.4
9	Absence of some desired medicines (magistral, unpaid, mixture, etc) on the system or not being able to prescribe them	112	7.3
10	Not being able to correct, add, delete after the prescription has been sent	74	4.8
11	Problems related with the medicine list	67	4.4
12	Not being able to prescribe some of the medical materials (catheter, diaper, etc)	45	2.9
13	Absence of desired medicine dosages on the system and not being able to prescribe them	29	1.9
14	Absence of some diagnoses in the system	18	1.2
15	Not being able to prescribe green prescription drugs electronically	17	1.1
16	Not being able to see the prescriptions that other physicians have written	6	0.4
	C- Problems experienced by patients	100	6.5
17	Problems related to the fact that the patient cannot see the written prescription	40	2.6
18	Problems giving equivalent medicine	22	1.4
19	Not being able to generate e-prescriptions for foreign patients and patients with private insurance	12	0.8
	D- Other	145	9.5
20	Being confused about some of the characters (Q, O, 0) in the prescription code and not being able read them	26	1.7
21	Being called by the patient or pharmacist to be asked to write the prescription and for the code of prescription	8	0.5
22	Not trusting e-prescriptions	8	0.5
23	Other^b^	103	6.7
	Total	1756	

^a^Since some physicians gave more than one answer to this question, the total of the percentage exceeds 100. ^b^Some of the complaints made were mentioned rarely in terms of frequency (by only 1 or 2 family physicians), and were therefore gathered under the title “Other.” Patients seeing physicians as responsible for waiting problems, leading to demotivation in physicians, and pain in right hand and neck due to computer use can be given as examples of complaints stated by only a few family physicians.

## Discussion

In this study, family physicians’ opinions on e-prescription were evaluated. The most frequently stated contribution of the e-prescription application was speeding up prescription writing and saving time. The most common complaints were system-induced problems and internet problems. Despite some problems with e-prescription practice, it was found that a significant portion of family physicians were satisfied with e-prescription. When analyzing the studies conducted in other countries on e-prescriptions, it was seen that the level of satisfaction with e-prescriptions is quite high. In the studies by Tan et al^8^ in Singapore and Jariwala et al^25^ in America, it was determined that 87% of physicians and 83% of e-prescribers were satisfied with the application of e-prescriptions, respectively. In studies conducted in Sweden,^26^ Austria,^27^ and England,^7^ it was found that a significant proportion of physicians think that the application of e-prescriptions is beneficial. Moreover, in a study by Gider et al^17^ with 248 physicians in Turkey, 62% of the physicians supported the application of e-prescriptions.

All of the factors in Table 3 can be interpreted as the reasons why the family physicians were satisfied with the application of e-prescriptions, the most important factor of physicians’ satisfaction was the fact that e-prescriptions can be generated faster than manual ones. More than 1 in 3 physicians (36.6%) indicated that e-prescriptions speeded up prescription writing and saved time. According to this, the application of e-prescriptions shortens the prescription writing time and thus saves time to family physicians, and this time can then be used for other work and processes (eg, patient examination, etc). In a study by Gimenes and Miasso^28^ in Brazil with 84 health professionals, it was shown that among the biggest advantages of e-prescriptions was the fact that they can be generated faster. Other factors that help physicians save time are removing the handwriting; being able to see medicines formerly prescribed to the patient, medicine equivalents, and prices written on the system, which speeds up the physician’s decision-making process regarding which medicine to prescribe; simplifying corrections to the prescription; being able to make additions to the prescription; and simplifying the addition of explanations.

In this research, it was found that a family physician writes an average of 47 prescriptions per day, and writing an e-prescription takes 3 minutes. According to this, family physicians allocate an average of about 140 minutes (47*3 = 141) per day to write prescriptions. It is known that family physicians work 8 hours per day. Therefore, family physicians spend an important part of their time (29.4%) generating prescriptions. When one considers that family physicians do not prescribe medicines for some patients but instead conclude the patient examination with counselling or a referral, it can be seen that the number of patients seen in 1 day by a family physician is higher than the number of prescriptions written daily (mean = 47.2). Considering the high workload of family physicians and the amount of time spent writing prescriptions, it is an important feature of e-prescriptions that they can be generated faster than manual ones.

With regard to the positive effects of the application of e-prescriptions, saving on paper and toner are among the issues that the family physicians point out most frequently. A total of 28.6% and 14.3% of the family physicians in this research indicated that e-prescriptions provided savings on paper and toner, respectively. According to this, the application of e-prescriptions has important positive effects also in financial terms. Furthermore, in different studies e-prescriptions have been found to be more cost-effective than paper prescriptions.^29^

In this study, 9.3% of the family physicians pointed out that the application of e-prescriptions reduced errors in prescription writing, e-prescriptions were legible, exact, and complete, provided convenience to the pharmacist in reading the prescription, and thus reduced errors of giving the incorrect medicine or dosage. Additionally, in the study by Gimenes and Miasso^28^ the errors in generating prescriptions have reduced with the application of e-prescriptions. In the study by Gider et al^17^ nearly half of the participating physicians were found to believe that the application of e-prescriptions made a positive contribution to patient safety. As physicians have a tendency to write prescriptions as soon as possible due to their high workload, problems arise in relation to the information written on the prescription not being exact or legible, such as medicine, patient’s name, diagnosis, date of prescription, dosage of prescribed medicine, and daily dosage. Missing information on the prescription or failure in reading manually written prescriptions correctly might lead to returning the prescription to the physician. In this situation, the physician might need to allocate time for rewriting the prescription, thus leading to an increased workload, patient shuttling between the pharmacy and the healthcare facility, loss of time, and arguments between physician and patient, and physician and pharmacist. A percentage of the physicians in the research also indicated that e-prescriptions have reduced the arguments between patient and physician and between pharmacist and physician in relation to that issue. As e-prescriptions cannot be generated if there is essential information missing on the prescription, complete information is thus ensured. It can be said that prescriptions being exact and complete reduces relevant problems. Moreover, the illegibility of the name of the prescribed medicine on manual prescriptions may cause the pharmacist to give the incorrect medicine to the patient, which may cause important problems for the patient. Yorulmaz^30^ suggested that prescriptions that are not complete and legible may also lead to treatments that are not effective or safe, recurrence and prolongation (leading to chronification) of diseases, complications, and suffering of patients. Taking into consideration the issues associated with manual prescriptions, e-prescriptions seem to make important contributions to preventing these errors.

In our study, another issue raised by family physicians regarding the benefits of e-prescriptions was being able to see previously prescribed medicines and reports provided to the patient, and the simplification of accessing this information. The fact that physicians can easily access information on previous medicines and tests in the system might provide the chance to pre-assess a treatment to be prescribed, in addition to preventing the repetition of tests.

The family physicians in the present study drew attention to the benefits of the application of e-prescriptions in terms of patients and pharmacists. In previous studies, it was found that not only physicians, but also patients and pharmacists were satisfied with the application of e-prescriptions.^31,32^ Reducing patient waiting time, creating the perception of a high-quality service, removing the problem of tearing or loss of paper prescriptions, reducing medicine or dosage errors, simplifying the process for patients to obtain their medicines, and general increase in patient satisfaction can be counted as benefits for the patient. E-prescriptions being exact, complete, and legible, and reducing arguments with physicians over prescription writing can be considered benefits of the application of e-prescriptions for the pharmacist.

In spite of the aforementioned positive contributions of the application of e-prescriptions, some related problems have been identified. The most common complaints of the family physicians were failure to obtain an internet connection, slowness of the connection, disconnection, and failure to obtain mobile internet services. Another problem related to the internet was dependency. It is necessary to have an internet service to upload (forward) the generated e-prescription to the system, to see the patient information in the system, in brief, to be able to generate an e-prescription. For this reason, it is a necessity to establish a strong internet infrastructure in order to be able to implement e-prescriptions reliably.

Additionally, failure of the computer (hardware) and insufficiency of the infrastructure were identified in this study among the problems encountered in the application of e-prescriptions. In the research by Gimenes and Miasso^28^ dependency on computers was indicated as one of the disadvantages of e-prescriptions. As e-prescription generation takes place in an electronic environment, a computer is needed. Therefore, it is necessary that computers available in healthcare facilities have appropriate equipment (sufficiency) and their maintenance be performed regularly.

Loss of time and waiting were found to be among the most common complaints in this study. Slowness or failure in obtaining an internet connection, failure in functioning of computer (hardware), failure in functioning of the program (software) developed for e-prescribing, and the user lacking information may cause loss of time. Therefore, in addition to providing a reliable internet service and ensuring that computers and the e-prescription program function correctly, it can be useful that users be trained in generating e-prescriptions. Craxford et al^33^ found in their study that providing training to new physicians in the application of e-prescriptions reduced errors in e-prescription writing and speeded up the process.

Additional problems with the application of e-prescriptions found in this study were failure in generating e-prescriptions for foreign patients and patients with private insurance, failure in prescribing some medical equipment, some medicines not being available or not being able to prescribe them, the desired dosage not being available or not being able to prescribe it, failure in prescribing green prescription drugs, existence of medicines in the system that are not available on the market, not being able to see prescriptions written by other physicians, unpaid medicines not being stated, and not being able to make corrections, additions, or deletions after sending an e-prescription. Thus, it is necessary that the above problems be dealt with by technical experts that take charge of the launching and implementation of e-prescribing, so that it can be re-designed in line with the needs of physicians.

The study results are limited to the evaluations of the participating family physicians. However it was shown that the participants was broadly representative of the family practitioner population. Since this study was carried out via e-mail, the number of questions were kept low and several socio-demographic characteristics of the participants were not asked. Open-ended questions, however, provided in-depth information on family physicians’ experiences with the effects of e-prescription. Future studies with more questions, including location of the physicians, may be conducted by other survey methods to gain more insight. In-depth interviews may be conducted with fewer physicians to be identified by stratified sampling according to geographical distribution. It is also suggested that further research be conducted with other parties involved in the application of e-prescriptions such as patients, pharmacists, and administrators in order to extend the scope and the validity of the results of this research.

## Conclusion

When the statements of the family physicians about the benefits and problems related to the application of e-prescriptions are considered together, it can be seen that some statements are counted as both benefit and complaint. Statements such as saving time/loss of time, simplifying the correction of prescription/not being able to make correction on prescription, and e-prescriptions being safe/not trusting e-prescriptions are examples illustrating this point. It is understood from this, some problems experienced in the application of e-prescriptions (eg, problem of waiting) become an advantage (eg, facilitation of prescription writing and saving time with the application of e-prescriptions) in a well-functioning implementation of e-prescriptions. Moreover, the mean level of satisfaction of the family physicians that did not experience problems with the application of e-prescriptions, was found to be higher in comparison to that of those who did experience problems. According to this, by eliminating the problems encountered in the application of e-prescriptions, the level of related satisfaction might increase.

## Acknowledgements

The authors wish to acknowledge the contribution of the family physicians who participated in this study.

## Ethical issues

Official approval were obtained from the Public Health Institution of Turkey.

## Competing interests

Authors declare that they have no competing interests.

## Authors’ contributions

SB: Conception and design, acquisition of data, analysis and interpretation of data, drafting of the manuscript, critical revision of the manuscript for important intellectual content, administrative, technical, or material support. AY: Analysis and interpretation of data, drafting of the manuscript, critical revision of the manuscript for important intellectual content, statistical analysis, administrative, technical, or material support. SK: Conception and design, analysis and interpretation of data, drafting of the manuscript, critical revision of the manuscript for important intellectual content, administrative, technical, or material support, supervision.

## Authors’ affiliations

^1^Department of Health Care Management, Faculty of Health Sciences, Hitit University, Çorum, Turkey. ^2^Program of Health Institutions Management, Vocational School of Health Services, Batman University, Batman, Turkey. ^3^Department of Health Care Management, Faculty of Economics and Administrative Sciences, Hacettepe University, Ankara, Turkey.

## Key messages

**Implications for policy makers**

- Prior to the implementation of the e-prescription system nationwide, it is important that the technical infrastructure is completely established and the integration between institutions is fully achieved.
- Evaluating the opinions of users after a pilot study of the e-prescription system will help prevent problems that may arise after the application has become widespread.
- E-prescribing provides important contributions in the prescribing process, especially in terms of saving time and eliminating handwriting errors. In addition, e-prescription has important contributions to patient safety and medication safety.

**Implications for public**

E-prescribing is a useful application for patient safety and satisfaction. Because it eliminates medication errors resulting from illegible handwritten prescriptions, shortens the prescribing time and makes it easier for patients to get medication from pharmacy.
